# Category Relevance Attenuates Overshadowing in Human Predictive Learning

**DOI:** 10.1037/xan0000357

**Published:** 2023-07

**Authors:** José A. Alcalá, José Prados, Gonzalo P. Urcelay

**Affiliations:** 1 School of Psychology, University of Nottingham; 2 Departamento de Psicología Experimental, Procesos Cognitivos y Logopedia, Universidad Complutense de Madrid; 3 School of Psychology, University of Derby

**Keywords:** overshadowing, pretraining, elemental, configural, intracategory

## Abstract

In situations in which multiple predictors anticipate the presence or absence of an outcome, cues compete to anticipate the outcome, resulting in a loss of associative strength compared to control conditions without additional cues. Critically, there are multiple factors modulating the magnitude and direction of such competition, although in some scenarios the effect of these factors remains unexplored. We sought to assess whether the relative salience of the elements in a compound of cues modulates the magnitude of the overshadowing effect in human predictive learning. Two separable categories (i.e., colors and symbols) were used in a predictive learning task. In Experiment 1, different groups of participants were granted with different time of exposure to a compound of cues belonging to different categories (color and symbol) to evaluate potential differences in the magnitude of overshadowing. Furthermore, we used posttest questionnaires to assess whether participants used either only one or both categories during training, and assessed if this impacted the magnitude of overshadowing. In general, overshadowing was not modulated by the time of exposition, except in the case of very short time of exposition with prominent learning about the most salient category. In Experiment 2, the relative salience of a category was biased via prior experience either with a biconditional discrimination or attending only the relevant category (either color or symbol). The previously relevant category was less prone to overshadowing, but not the alternative one. Results are discussed in light of attentional and configural theories of associative learning.

In standard Pavlovian conditioning, pairing a single conditioned stimulus (CS) with an unconditioned stimulus (US) typically results in the development of a conditioned response (CR) to the CS. In the basic scenario in which the target cue (X) is paired repeatedly with an outcome, X acquires strong behavioral control; however, if during training X is accompanied by another cue (A—forming the compound AX), behavioral control by X is reduced compared to when X is trained alone; this is an instance of the overshadowing effect (Pavlov, 1927). In a similar scenario, when an organism receives additional training with A, before or after training with the compound AX, this results in a further reduction in the response to X, because A blocks learning or performance about the target cue (Kamin, 1969). Therefore, in the presence of multiple predictors of a given outcome, organisms tend to select between them, favoring one event while discounting others. One of the key attributes of the stimuli forming a compound that modulates competition is their salience (Mackintosh, 1976).

Salience is a fundamental property of stimuli that affects learning and is often represented in formal models of learning through a specific parameter, such as the alpha in the well-known Rescorla–Wagner model (Rescorla & Wagner, 1972). This parameter modulates the speed of learning in a linear manner when the subject learns about a single stimulus: learning proceeds more readily with highly salient than with relatively low salient stimuli. In the case of compound learning, if one of the elements of the compound is more salient, such as a loud tone, it can overshadow the less salient element, such as a mild white noise. Consequently, the more salient element accrues greater behavioral control than the less salient one (Mackintosh, 1976). Interestingly, when both stimuli are equally salient, they recruit similar levels of behavioral control (see Mackintosh, 1976, Experiment 2), mitigating to some extent the overshadowing effect.

In the case of blocking, studies conducted in nonhuman animals (Feldman, 1975; LoLordo et al., 1982) and humans (Denton & Kruschke, 2006) revealed that the magnitude of blocking decreases when the salience of the blocked cue is increased (i.e., they observed somewhat similar levels of CR to the blocked compared to the control cue). However, this effect has not been consistently replicated in humans. For example, Le Pelley et al. (2014) conducted a study in which they manipulated the semantic salience of the cues in the context of causal attribution learning using the allergy task, expecting that cues of higher semantic salience (e.g., snake) would be more resistant to blocking than target cues of low semantic salience (e.g., cucumber). However, they found the opposite pattern of results: when the blocked cue was of higher salience (e.g., snake), the difference between the blocked and the control cues was larger (i.e., more blocking) than when using lower salience target cues. Unsurprisingly, they found that when cues were trained alone, the high salience cues received higher ratings, in accordance with the idea that higher salience recruits more behavioral control. In a category learning paradigm, Murphy and Dunsmoor (2017) observed that a salient, aversive critical feature did not reduce learning of other features relative to a control condition (but see Lau et al., 2020 for an example of overshadowing in category learning). Taken together, the results reviewed here suggest a differential effect of salience in human learning depending on task and parameter particularities. As far as we are aware, the effect of cue salience manipulations on overshadowing effect has not been assessed in human predictive learning, which is the focus of the work reported here.

Additionally, several studies have reported puzzling results indicating that the relative salience of the elements of a compound can result in either facilitation or potentiation. For example, training a target cue in compound with a more salient cue has been found to potentiate learning about the target compared to a control cue in taste aversion learning (Bouton et al., 1987; Durlach & Rescorla, 1980), fear conditioning (Urcelay & Miller, 2009), and spatial learning (Pearce et al., 2006). These studies suggest that the relative salience of the cues is important for integrating the elements of the compound and this may lead to either competition or facilitation. This is particularly intriguing, because predicting whether competition or facilitation is to be observed has proven challenging for learning theories (see Urcelay, 2017 for a discussion). However, all these studies mentioned above were conducted with nonhuman animals and little evidence is available on the importance of relative salience in human studies. At their best, studies using human participants have identified the specific conditions of temporal and spatial contiguity under which competition or facilitation is more likely to be observed (Alcalá, Kirkden, et al., 2023; Alcalá, Miller, et al., 2023; Glautier, 2002; Herrera et al., 2022). This advises that more research is needed to achieve a better understanding of the specific factors underpinning cue interaction phenomena in human participants.

It is worth pointing out that stimulus salience is not merely determined by its physical properties. According to selective attention theories of learning (e.g., Mackintosh, 1975), the stimulus salience, and consequently the degree of attention they receive, depends directly on its predictive value. According to Mackintosh, organisms tend to attend to and ignore relevant and irrelevant stimuli. For instance, in a blocking design, the blocking cue (A) is first paired with an outcome and, in a subsequent phase, a second cue is added to A to form the compound AX paired with the same outcome. According to Mackintosh’s (1975) theory, the animals will favor attending to the most relevant (predictive) element at the expense of the relatively poor predictors of the outcome. Given that A is already a good predictor, attention to the added cue X can be expected to decrease and be eventually ignored (e.g., Kruschke et al., 2005). In the case of overshadowing, given that both elements of the compound (A and X) share the same predictive value, the relative salience of each element determines the attention they receive, with more salient cues receiving increasing levels of attention at the expense of the less salient cues, which will be gradually ignored as compound training progresses (Mackintosh, 1976).

Enhanced attention is not specific to the predictive stimulus. There is evidence suggesting that increased attention can extend to a whole set of stimuli belonging to the same category as the stimulus trained as predictive. The literature on intradimensional versus extradimensional shifts exemplifies this. For example, Mackintosh and Little (1969; also see Durlach & Mackintosh, 1986 for similar results) trained pigeons to solve multiple discriminations. In the first stage, some animals were trained in a color discrimination whereas other animals were trained in a formally equivalent task in which different line orientations signaled the presence (or absence) of a reward. In a second stage, all animals were trained in a new discrimination using new colors as cues. They observed that pigeons in the intradimensional shift (color to color) learned the new discrimination faster than those in the extradimensional shift condition (line orientation to color). In line with Mackintosh’s attentional theory (1975), this result suggests that pretraining a category—making it relevant to predict the presence of a reward—increases the salience of the cues of that category based on their predictive value, and this transfers to new stimuli belonging to the same category. The facilitation observed in the intradimensional shift has been consistently observed in the literature (see Prados & Sansa, 2002 for an example in the spatial domain; and Tait et al., 2018 for a review) and serves as a basis for the notion that learned predictiveness can result in increases in attention—or salience to a category of cues.

Additionally, it has often been observed that previous experience solving a discrimination in which a configural solution is necessary, facilitates solving a second discrimination with a configural requirement (Glautier et al., 2016; Mehta & Russell, 2009). Therefore, previous experience solving a discrimination modulates subsequent behavior (i.e., transfers) concerning new discriminations (Urcelay & Miller, 2010). In the case of cue-competition phenomena, it is worth to mention that experience with a particular set of learning conditions can modulate the magnitude of subsequent competition. For example, Williams et al. (1994) only observed blocking when participants received initial pretraining favoring elemental processing, suggesting that elemental processing was critical to observe competition between cues. However, when participants engaged in a configural processing, either by the default structure of the task or by explicit previous configural training, blocking was not detected. Similarly, rats trained to solve a configural task subsequently showed attenuated overshadowing using a standard Pavlovian task (Urcelay & Miller, 2009). However, the study of category transfer has received little attention in the case of cue-competition phenomena in humans. Kaminski et al. (2008) found that making a particular cue relevant in a blocking design made other elements of the same category-relevant; hence other cues belonging to that category were more likely to be considered good sources of information, facilitating blocking of cues from an alternative category. Similarly, Buckley et al. (2014, 2015) found that, in a spatial learning task, prior experience with a particular category, either landmarks or the geometry of the arena, biased the navigational strategy used in different environmental conditions made up of new instances of stimuli belonging to the same categories (a new shape of the arena with different landmarks). That is, when in the first stage participants learned that landmarks (but not geometry) were relevant to find a goal, they persisted using landmarks in detriment of the geometry in a subsequent phase to navigate toward a goal in the presence of a new set of landmarks and geometric cues. The opposite pattern was found when geometry was relevant during the first phase, revealing that prior experience with either category was critical in shaping the navigation strategy subsequently adopted. The work reported here aims to assess this type of manipulation in the case of overshadowing in human predictive learning.

In the experiments reported below, we assessed compound conditioning in human predictive learning using a task in which a compound of two separable categories (a color and a symbol) are paired with an outcome. We used separable dimensions that are likely to be processed independently in contrast to integral dimensions—such as brightness and saturation—which cannot be processed independently and consequently recruit automatic holistic processing (Garner, 1976). The reason to use separable categories was to disentangle the role played by each category in determining the magnitude of the overshadowing effect. The predictive value acquired by the elements of the compound was compared (within-subjects) to the predictive value acquired by control cues trained alone belonging to the same category (i.e., we compared a color trained by itself with a color trained in compound with a symbol; and a symbol trained in isolation with a symbol trained in compound with a color). We anticipated the color category would be easier to process than the complex symbols used. If this assumption is correct and the color category has higher salience than the symbol category, it would be easier to learn about the color than about the symbols. Moreover, Experiment 1 manipulated the amount of exposure to the cues, with the goal of evaluating whether the relative salience of the elements forming the compound interacted with the amount of exposure. Because participants were trained with stimuli belonging to two separable categories, it could be possible that some participants spontaneously processed both categories while others focused on just one of them (either the color or the symbol). Following a procedure similar to the one previously used in human predictive learning (Ahmed & Lovibond, 2019; Lee et al., 2018; Lovibond et al., 2020; Wong & Lovibond, 2018), upon completion of the task we asked participants which strategy they used: whether they used the color, the symbol or both categories to solve the task. The participants’ declared strategy might influence the way the elements of the compound are processed, affecting the magnitude of the overshadowing effect. Experiment 2 aimed to investigate whether prior training with discriminations (using different stimuli but belonging to the same categories used in overshadowing) that require processing both categories (which presumably could equal their salience) or just one of them (increasing only the salience of either the color or the symbol category) would affect the magnitude of the observed overshadowing effect.

## Experiment 1

**Figure 1 fig1:**
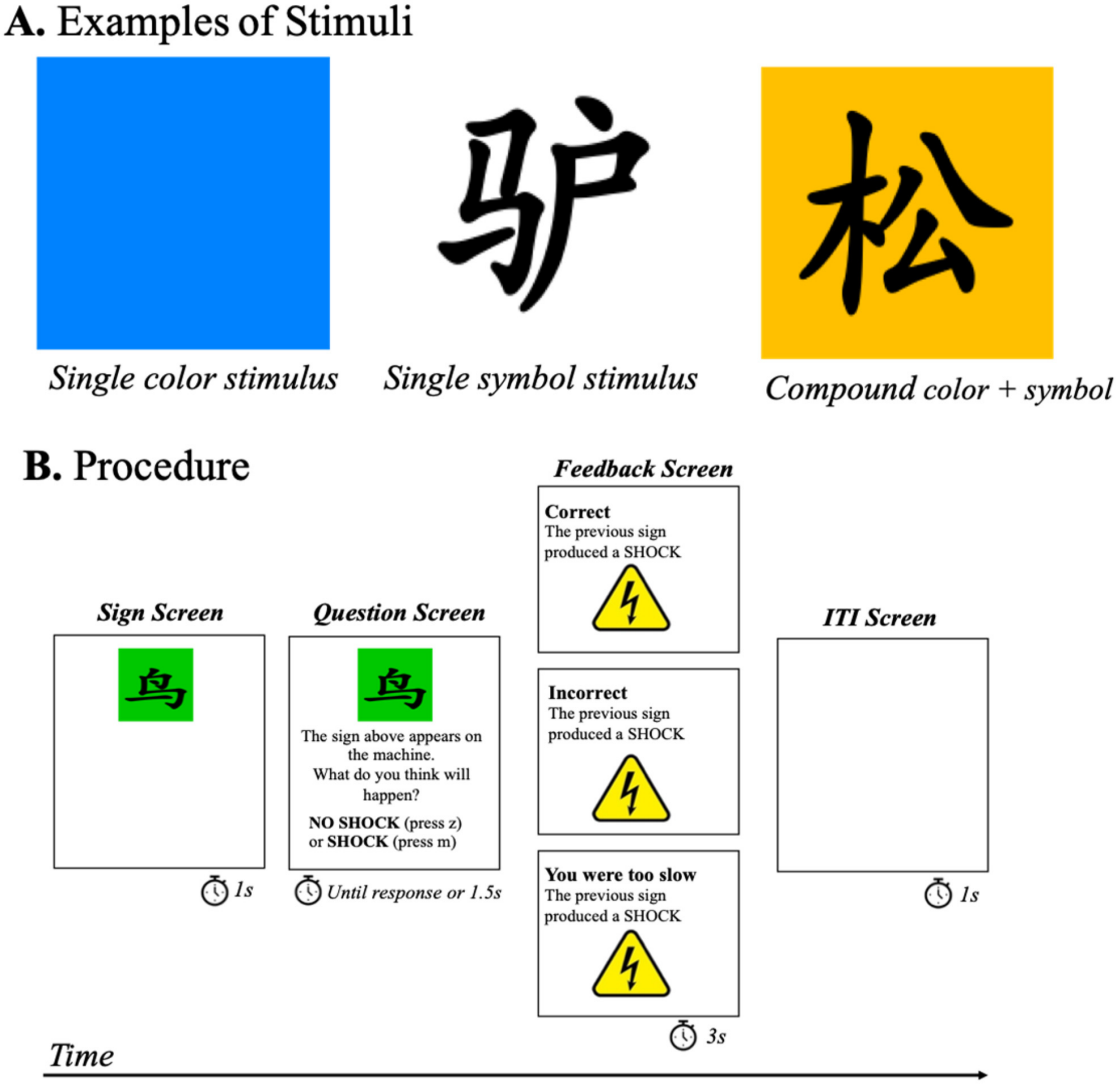
Stimuli and Procedure of Experiments 1 and 2 *Note*. Panel A represents the stimuli used during the task. Panel B symbolizes the procedure of the task. ITI = intertrial interval. See the online article for the color version of this figure.

Experiment 1 was run to assess how participants learn to predict an outcome using a color cue, a symbol cue, and a color–symbol compound in a within-subjects design (see Table 1). We anticipated that the cues trained alone will acquire higher predictive value than those trained as part of a compound (i.e., overshadowing effect). Stimuli belonging to both categories (i.e., compound) were presented simultaneously and formed a unique cue, with the symbol displayed over the color in the same physical space (see Figure 1A). The first category, symbol, was made of black Chinese characters; the second one was made of different colors. We anticipated that colors would be easier to process than the complex symbols (participants with no prior knowledge of Chinese were recruited). Colors are used in all the aspects of our daily life and they are processed automatically, requiring little effort to process different colors and discriminate between them. The colors used in our task were highly discriminable (without subtle differences along a specific category of the color like hue or luminance). Moreover, there is evidence that color is the most important category controlling behavior of nonhuman animals and humans in biologically relevant tasks (Kazemi et al., 2014; Sherratt et al., 2015). In terms of learning, for example, pigeons trained to predict outcomes based on colors and orientation lines showed an advantage to learn cues in the color category (Mackintosh & Little, 1969). On the contrary, complex symbols are more demanding, forcing participants to focus on specific details to discriminate between them. Such differences presumably result in two separable categories independent from each other. Control cues for each category (color and symbol) were also trained, enabling us to simultaneously assess overshadowing in both categories (see Table 1).[Table tbl1][Fig fig1]

In addition to using two categories, color and symbol, with different salience or effectiveness (as argued above), we also explored whether the length of exposure to the cues affects the magnitude of the overshadowing effect. Three groups of participants were given short (1s), medium (3s), or long (9s) exposure to the relevant cues (colors, symbols, and color–symbol compound). We conjectured that the amount of time exposure to the compound would determine the way in which participants integrate the information relative to each element of the compound. With a short exposure, participants may just focus on determining the relationship of the most discriminative and salient category, the color, neglecting the cue belonging to the alternative category, the symbol. However, longer exposure may allow adequate processing of both categories, allowing the integration of the symbol cues. Previous work suggests that long stimuli attenuate overshadowing in rats (Sissons et al., 2009) and reduce overselectivity in humans (Reynolds & Reed, 2018). However, we are not aware of studies exploring whether the signal length is a factor determining overshadowing in humans. In a recent experimental series from our laboratory (Herrera et al., 2022), we did not observe reduced overshadowing with longer CSs; however, this was based on a comparison across experiments, so it should not be considered as conclusive evidence for the absence of an effect of stimulus length in overshadowing.

Assuming that competition is the default outcome when facing a compound of two stimuli, an overshadowing effect was expected. However, with short exposure, given the anticipated salience imbalance, the color was expected to be more effective in overshadowing the symbol than the other way around. On the other hand, long exposure to the compound might allow a more efficient processing of the symbol category, contributing to equate the salience of the color and symbol categories. With similarly high salient competing stimuli, an attenuation of overshadowing could be expected (Mackintosh, 1976, Experiment 2). As an alternative, long exposure might allow for the processing of the compound as a configuration. A bias to encode the compound configurally in the long-exposure group (9s) might therefore attenuate cue competition (Urcelay & Miller, 2009; Williams et al., 1994).

In line with what Lovibond et al. (2020) have done in generalization experiments, we were also interested in the spontaneous strategies declared by participants after training with the compound color–symbol cues. In an overshadowing paradigm, the two elements of the compound CS have in principle equal opportunities to become associated with the outcome, although as mentioned above, this is influenced by the salience of the stimuli (Mackintosh, 1976). However, participants might develop a spontaneous preference for one of the elements of the compound and attend exclusively to that category—increasing its salience. Alternatively, they could try to process both elements of the compound, which would lead to the development of predictive value by both elements and, therefore, an increase in their salience. To the best of our knowledge, the spontaneous strategy declared by participants following the completion of the task has not received much attention in compound training phenomena such as the overshadowing effect. To evaluate the strategy declared by participants, we included a forced-choice question at the end of the experiment (see Lovibond et al., 2020). If participants declared only processing one category (either the color or the symbol), we may expect an asymmetrical overshadowing, since the spontaneous preference for the color might prevent the processing of the alternative category (symbol). In that case, given that the symbol is ignored, the color would enter into association without competition and no difference should be expected with the color trained by itself (the control condition): the preferred category (the color) overshadows the alternative category, but not the other way around. The same might apply for the spontaneous preference for the symbol. In other words, spontaneous preference for one category could result in asymmetric overshadowing. However, because competition is the most likely scenario, when processing both categories overshadowing could still be observed. However, this strategy involved a configural strategy which, as pointed out above, presumably attenuates competition (e.g., Urcelay & Miller, 2009). Finally, the type of strategy may be related to the time of exposure to the cues. We anticipated that participants in Group 1s would show tend to declare they used the color, whereas with longer exposure (9s) participants might tend to declare a predominant use of both cues.

To recap, in Experiment 1, participants were exposed to a discrimination in which different colors and symbols were used as signals in a predictive learning task. Compounds were formed by a color and a symbol, and for each category, there was a specific control cue trained alone in a within-subjects design (see Table 1). Three different groups received different lengths of exposure to the cues during training (1s, 3s, or 9s). We expected attenuated overshadowing with increased exposure to the cues. Finally, we explored whether the magnitude of overshadowing was modulated by the strategy declared by participants in a posttest forced-choice question.

### Method

#### Participants

One hundred and twenty participants (77 female, 39 male, two nonbinary, and two preferred not to say) were recruited from Prolific. Their mean age was 36.26, *SD* = 11.88 (range 21–67). We used similar studies conducted online and exploring overshadowing as a reference to estimate the sample size (*n* = 40; Alcalá, Miller, et al., 2023). All participants were fluent in English language and had completed more than 200 prior approved tasks in Prolific with a mean approval equal to or greater than 98%. An explicit exclusion criterion was included to prevent people with knowledge of the Chinese language to participate. Participants could take part in this series only once. These criteria were applied in this and the next experiment. Participants received compensation for their contribution based on the time it took each experimental group to complete the task (£1.5, £1.75, or £2.5 for Groups 1s, 3s, and 9s, respectively). The study was approved by the Ethics Committee at the University of Nottingham.

#### Design

A 3 (group: 1s, 3s, 9s) × 2 (category: color vs. symbol) × 2 (cue: control vs. target) mixed-design was used. The group factor was manipulated between-subjects, and each group experienced the stimuli for a fixed amount of time, either 1s, 3s, or 9s. The other factors were manipulated within-subjects. For the factor category, there were two categories of stimuli: color and symbol. Color category was represented by a square of several colors and the symbol category by using different Chinese characters. The factor cue had two levels: control refers to the cue trained alone (in both categories) and target to the cue trained in compound with a cue of the alternative category. For example, as the upper part of Table 1 shows, for the category color, the control cue A was trained alone and the critical comparison is with the target cue C (trained in compound with cue X—a symbol). Note that this design allows a control cue in each category (A and V) to be compared with the corresponding target cue in each category trained in compound (CX): the color A is compared to the color C whereas the symbol V is compared to the symbol X. The same applied to the nonreinforced cues, trained alone (B, W) or as part of a compound (DY).

#### Materials and Apparatus

The task was inspired by Lovibond et al.’s (2020) task. The experiment was programmed and hosted online using the Gorilla Experiment Builder (Anwyl-Irvine et al., 2020). Two set of stimuli were used, colors and symbols. A colored square (Figure 1A) of approximately 300 × 300 pixels and a black Chinese character (see Figure 1B) of approximately 200 × 200 pixels over a white background were used as stimuli. When stimuli appeared as a compound, the symbol was presented over the colored square (see Figure 1A). As colors we used: green (RGB: 90, 197, 58), blue (RGB: 56, 128, 247), orange (RGB: 245, 195, 66), pink (RGB: 234, 51, 157), and purple (RGB: 104, 52, 154). As symbols we used the characters for animals: bird (鸟), donkey (驴), squirrel (松), raccoon (狸), and kangaroo (袋). These stimuli were counterbalanced across subjects.

#### Procedure

After reading and signing the consent form, participants were presented with the following instructions:

*[Screen 1] Please, read the instructions for the task carefully. After reading the instructions, you will be asked two questions to make sure you have understood them. If you fail to respond correctly to all questions, you will have the chance to read the instructions again. You will not be able to start the experiment until you respond correctly to all questions.*

*[Screen 2] We would like you to imagine that you have come across a strange machine. It appears to have a display on it, as well as poster that says “WARNING: this machine gives electric shocks!! When you see warning signs like this ______, do NOT touch!”*

*Unfortunately, the area of the label that shows the warning signals has been scratched off, so you do not know which signs predict danger. Your job is to work out what kinds of signs on the machine predict the shock.*

*[Screen 3] The experiment is composed of a number of “trials.” On each trial, you will be presented with a sign on the shock machine. You will then make a prediction about whether you think a shock will occur. In the FIRST phase of the experiment, you will receive feedback for your predictions about whether a shock occurred or not. In a final phase, you will need to rate on a scale to what extent you think each sign caused a shock, but you are not going to receive feedback. You will receive further instructions at the beginning of this phase.*

*[Screen 4] In the first phase, you will learn which signs lead to shock. We will present the same signs to you MULTIPLE times. On each trial, the sign will appear on the screen and next, the question “The sign above appears on the machine. What do you think will happen?” Press “z” if you think NO SHOCK will occur, or “m” if you think a SHOCK will occur. You need to wait for the question to appear to make your decision. From the moment the question appears you need to respond quickly. You will have 1.5 s to respond. You must respond within this time. If you do not respond during this time, you will receive feedback, however, your response will not be recorded. Look at the signs and the feedback carefully. Use this feedback to find out which signs lead to shock. Don´t worry, at first you will have to guess because you do not know much about these signs, but eventually you will learn which sign leads to shock and you will be able to make the correct predictions. If you are not able to respond, you still will receive feedback, however, your response will not be recorded. Please, try to avoid to the best of your abilities trials without a response.*

After the instructions, a couple of forced-choice questions were introduced to check whether participants understood the instructions. In case participants failed any of the questions, they had to read the instructions again until they correctly responded to both questions. The questions and answers were:

1 *What are you instructed to do during the task?*
  • *To evaluate whether or not different signs predict a shock.*
  • *To respond as fast as possible when you see a sign.*
  • *To evaluate how beautiful are the signs to you.*
2 *What is the key to select “no shock?”*
  • *z*
  • *m*
  • *Spacebar*

Once participants had correctly responded to these questions, the training phase started. On each trial, the stimuli appeared for 1s/3s/9s depending on the experimental group (see Figure 1B). After the respective time for each group had elapsed, the question “What do you think will happen?” NO SHOCK press “Z” or SHOCK press “M” appeared on the screen (the stimuli remained on the screen). The question and stimuli were presented either until the participant responded or for a maximum of 1,500 ms—the time window to register the response. Following the response or time limit the screen displayed corrective feedback (“correct”; “incorrect”; or “too slow” in those trials in which the participant failed to respond within the 1,500 ms time window) and information on whether the outcome was present (“the previous sign produces a shock”—with an image of a virtual shock) or absent (“the previous sign did not produce a shock”). Feedback was displayed for 3s in the absence of the stimuli, followed by a 1,000 ms intertrial interval (ITI) during which a white screen was displayed. The ITI was kept constant in all groups to avoid benefits in terms of spacing of trials.

The training phase consisted of eight presentations of each cue (see Table 1). Training was divided into four blocks of 16 trials with two presentations of each cue per block (64 trials in total). The order of trials within each block was randomly determined without any restrictions. For the color category, two cues were presented alone, one reinforced (A+, the control cue), and another not reinforced (B−). Similarly, for the symbol category, two cues were presented alone, one reinforced (V+, the control cue), and another not reinforced (W−). Two additional colors (C and D) were presented in a reinforced compound (CX+; C was the target cue for the color category and X for the symbol category) and as a nonreinforced compound (DY−). Reinforced and nonreinforced cues were always followed or not by the outcome according to their programmed contingency. The partial color (E±) and the partial symbol (Z±) cues received one reinforced trial and one nonreinforced trial per block. The partial cues were introduced to introduce some variability to avoid participants attention wandering during the task.

During the test phase, a single test question was presented with an image of each cue alone (each letter in Table 1). Participants read the following question: “What is the likelihood of the sign above leading to SHOCK?” There was a horizontal rating scale, ranging from 0 (*definitely no shock*) to 100 (*definitely shock*). The pointer was positioned at 50 on the scale, and participants could move the pointer in either direction with the mouse. There was no time limit to respond to each question. In a first block, each cue of the design was presented randomly. After that, a second block was conducted asking again about all cues in a random order. The average rating for both test questions for each cue was used as the final rating.

After the test phase, we introduced a final question asking participants about the strategy used in the experiment.

*We will now ask you some question about what you have learned in the experiment. Please note that this question refers to what you thought during the FIRST phase of the experiment (where you received feedback). During the first phase, there were some signs formed by a color square and a black symbol character. Please, read each option carefully and select the statement that you think is most true.*

Each statement was displayed in a separate box. The order of the boxes was different for each participant and there was no time limit to respond to this question.

*I use the combination of both, color and black symbol, to predict whether the sign produced or not a shock*

*I use mainly the black symbol to predict whether the sign produced or not a shock*

*I use mainly the color to predict whether the sign produced or not a shock.*

#### Transparency and Openness

We report how we determined our sample size, and we explain all data exclusions (if any), all manipulations, and all measures in the study. All data reported in these experiments are available at: http://doi.org/10.17639/nott.7254. Data were analyzed using IBM SPSS Statistics (Version 27) and JASP (2022). This study’s design and its analysis were not preregistered. The task was programmed using Gorilla, and the materials are available upon request.

#### Data Exclusion

In all experiments, we conducted several checks to ensure data quality: participants who declared being color-blind were eliminated; during training, participants who failed to respond in more than 20% of the trials were removed from the analyses; at the end of training, only participants with higher average ratings to both cues presented alone and reinforced (A+ and V+) compared to the nonreinforced cues (B− and W−) were included in the analyses, otherwise they were not included. We asked participants if they had any knowledge of Chinese language; if they selected yes, they were removed from the analyses. Finally, at the end of the task, we asked participants about their subjective commitment during the task with the following question:

*Well Done! The experiment is over. Just one last question. Did you give your full attention to the experimental task (as opposed to sometimes doing other things like using your smartphone) while stimuli were being presented? Please, answer honestly; this question has no impact on your payment. There are two options below “Yes” and “No.”*

Participants who selected “No” were removed from the analyses and data of these participants were not replaced.

After applying these checks, 99 participants were considered for data analyses (32 in Group 1s, 32 in Group 3s, and 35 in Group 9s).

#### Data Analyses

##### Training

We focused on the last block of training trials. A 3 (group: 1s, 3s, 9s) × 2 (reinforcement: reinforced vs. nonreinforced) × 3 (type: color, symbol, compound) mixed-ANOVA was conducted, with the first factor manipulated between-subjects and the other two within-subjects. The critical goal of these analyses was to check the differences between reinforced and nonreinforced cues and assess potential differences between groups at the end of training. We used the proportion of responses predicting the shock in each block as the index of learning.

##### Test

We analyzed the averaged ratings of all cues during the test phase to assess differences between both categories. Critically, an overshadowing index was calculated as the difference between the control cue and the target cue in the reinforced cues (control − target) in each category. This is an index of competition whereby a value of 0 means the absence of cue interaction (neither overshadowing nor facilitation). In the reinforced cues, positive scores are indicative of cue competition or overshadowing whereas negative scores would be indicative of cue facilitation or potentiation. In the nonreinforced cues, positive scores are indicative of facilitation, whereas negative scores would be indicative of overshadowing. A 3 (group: 1s, 3s, 9s) × 2 (reinforcement: nonreinforced vs. reinforced) × 2 (category: color vs. symbol) mixed-ANOVA was conducted. In subsequent analyses, one-sample *t* tests were used to corroborate whether the index was different from zero or not. We provided *BF*_01_ to test the reliability of the lack of overshadowing using the default Cauchy prior distribution (JASP, 2022). As a general guide, we considered the Bayes factor above 3 as substantial evidence for the lack of differences (Wagenmakers et al., 2011). The rejection criterion was set at 0.05 for all statistical tests. Partial eta-squared measures are presented for effect sizes, and their 90% confidence intervals were reported using the software available in Nelson (2016). Cohen’s *d* was provided for the *t* comparisons. When the assumption of sphericity was violated, the Huynh–Feldt correction was applied in the corresponding tests of main effects or interactions.

### Results

#### Overshadowing Training

**Figure 2 fig2:**
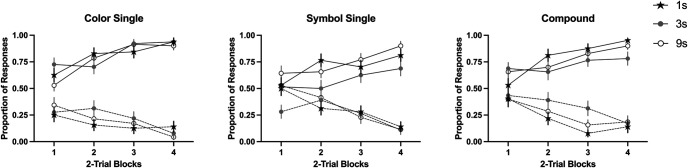
Results From the Training Phase *Note*. Proportion of participants who predicted the shock outcome in blocks of two trials. Each figure represents the response in the presence of the color cue alone (left), the symbol cue alone (middle), and the compound of color + symbol (right). Star symbols symbolize Group 1s, gray symbols Group 3s, and white symbols Group 9s. Solid lines represent the reinforced cues and dashed lines represent the nonreinforced cues. Error bars are *SEM*.

A 3 (group: 1s, 3s, 9s) × 2 (reinforcement: reinforced vs. nonreinforced) × 3 (type: color, symbol, compound) mixed-ANOVA in the last block of training revealed a main effect of reinforcement *F*(1, 96) = 827.47, *p* < .001, η_p_^2^ = .90, 90% CI [0.86, 0.92], a marginal effect of type, *F*(1.89, 181.45) = 3.08, *p* = .051, η_p_^2^ = .03, [<0.01, 0.08] and a significant Reinforcement × Type interaction, *F*(2, 192) = 4.73, *p* = .010, η_p_^2^ = .05, [0.007, 0.10]. However, the group factor was not significant, neither as a main effect nor in interaction with any other variable, largest *F* (for the main effect of group), *F*(2, 96) = 2.76, *p* = .068, suggesting that all groups performed similarly at the end of training. Subsequent analyses exploring the Reinforcement × Type interaction, revealed that the simple effect of type was not significant in the nonreinforced cues, *F*(2, 196) = 2.42, *p* = .092, but it was significant in the reinforced cues, *F*(2, 196) = 5.10, *p* = .007, η_p_^2^ = .05, 90% CI [0.008, 0.10], with lower ratings to the symbol cue compared to color cue, *F*(1, 98) = 8.88, *p* = .004, η_p_^2^ = .08, [0.02, 0.17], but not the compound cue, *F*(1, 98) = 3.15, *p* = .079, η_p_^2^ = .03, [0.001, 0.10]. In general, the symbol cues recruited lower rates of responding, in line with the idea that color cues are easier to learn. However, this effect was not modulated by the length of exposure (Figure 2).[Fig fig2]

#### Test Phase

Table 2 summarizes the ratings during the test phase in each group. All groups showed good discrimination between reinforced, nonreinforced, and partially reinforced cues. The lowest average rating for reinforced cues (A+, C+, V+, and X+) was above 50; in contrast, the largest rating for the nonreinforced cues (B−, D−, W−, and Y−) was below 45; finally, for the partially reinforced cues (E and Z) the ratings were somewhat in the middle. One relevant analysis results from the comparison of the two elements of the compound, that is, the target cue for symbol and color categories. In the case of reinforced cues, there was a main effect of category, with overall higher ratings to the color compared to the symbol, *F*(1, 96) = 9.43, *p* = .003, η_p_^2^ = .09, 90% CI [0.02, 0.19] but modulated by group factor, *F*(2, 96) = 4.09, *p* = .020, η_p_^2^ = .80, [0.24, 0.46]. Interestingly, only Group 1s showed differences between color and symbol categories, *t*(31) = 3.85, *p* < .001, this was not the case in Group 3s, *t*(31) = 1.43, *p* = .161 (*BF*_01_ = 2.09) nor in Group 9s, *t*(34) *=* 0.03, *p* = .974, (*BF*_01_ = 5.51). A similar pattern was observed with the nonreinforced cues, with differences in the case of Group 1s, *t*(31) = 5.43, *p* < .001, but not in the case of Group 3s, *t*(31) = 1.47, *p* = .151 (*BF*_01_ = 1.99), nor in Group 9s, *t*(34) = 0.65, *p* = .515, (*BF*_01_ = 4.51). Increasing the length of exposure to the cues resulted in the ratings between both elements of the compound being more similar, especially with 9s, with the Bayes factor above 3 for reinforced and nonreinforced cues. This is consistent with the notion that in the long exposure condition, the two cues (color and symbol) that form the compound have similar salience, overcoming the bias toward the color observed in the other two groups. However, this long exposure had little effect on the magnitude of overshadowing (see below).[Table tbl2]

**Figure 3 fig3:**
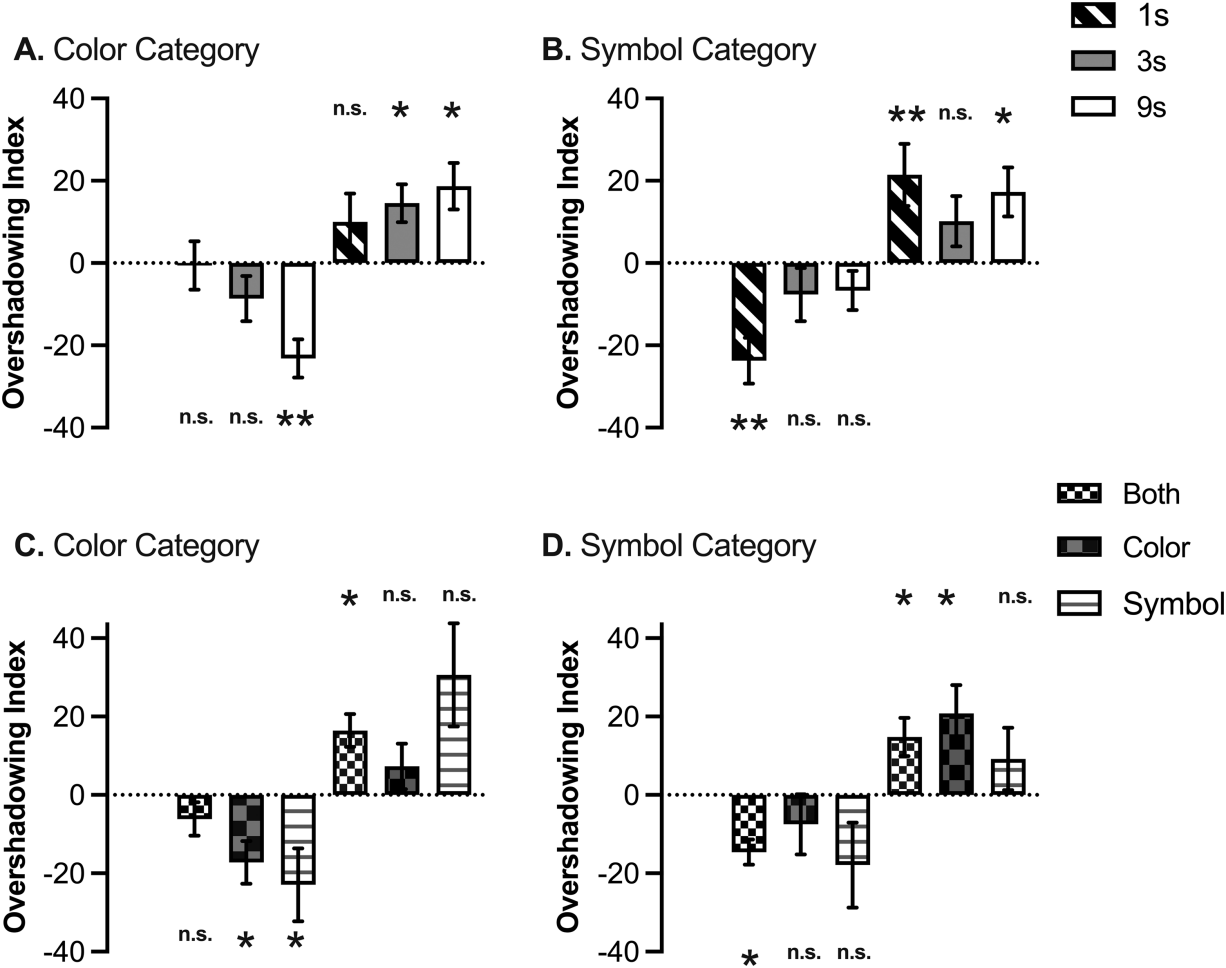
Overshadowing Index in Experiment 1 *Note*. Panels A and B display the overshadowing index of groups trained with a 1s, 3s, and 9s stimulus duration: Panel A displays the indexes for the color cues whereas Panel B displays the indexes for the symbol cues. Panels C and D display the overshadowing index of the participants according to their declared processing type (Both: using color and symbol; color; and symbol). Panel C displays the data corresponding to the color category whereas Panel D refers to the indexes of the symbol cues. Note that the overshadowing index was calculated by subtracting the predictive rating of the target cue (trained in compound with a competitor) from the ratings of the control cue (trained by itself); a value of 0 represents similar ratings to the control and target cues, that is, no overshadowing. Positive and negative values are indicative of overshadowing depending on the type of reinforcement. * *p* < .005. ** *p* < .001. n.s. *p* > .05. Error bars are *SEM*.

Key to our goal was the overshadowing index. Figure 3 illustrates the overshadowing index in both categories (colors and symbols) for nonreinforced cues (left side of each figure) and for the reinforced cues (right side of each figure). For the nonreinforced cues, values below 0 represent that the target cue received higher ratings than the control cue, this suggests cue competition or overshadowing. In the case of reinforced cues, values above 0 represent competition between cues, that is, the target cue trained in compound received lower ratings than the respective control cue trained alone in each category. In both scenarios, a value of 0 means that the target and the control cue received similar ratings.[Fig fig3]

Figure 3 suggests an overall overshadowing effect. In general, pooling data across groups confirmed such a tendency. One-sample *t* tests revealed a significant overshadowing for nonreinforced cues, *t*(98) = 3.49, *p* = .001, *d* = 0.35 (*M*_color_ = −11.18, *SD*_color_ = 31.83), and *t*(98) = 3.80, *p* < .001, *d* = 0.38 (*M*_symbol_ = −12.48, *SD*_symbol_ = 32.62) for color and symbol, respectively; in the case of reinforced cues, overshadowing was also reliable in both categories: color, *t*(98) = 4.37, *p* < .001, *d* = 0.44 (*M*_color_ = 14.53, *SD*_color_ = 33.08), and symbol, *t*(98) = 4.33, *p* < .001, *d* = 0.44 (*M*_symbol_ = 16.33, *SD*_symbol_ = 37.50).

However, Figure 3 (panels A and B) suggests subtle differences between groups. A 3 (group: 1s, 3s, 9s) × 2 (reinforcement: reinforced vs. nonreinforced) × 2 (category: color vs. symbol) mixed-ANOVA revealed a main effect of reinforcement, *F*(1, 96) = 54.27, *p* < .001, η_p_^2^ = .36, 90% CI [0.24, 0.46], and a triple interaction, *F*(2, 96) = 6.36, *p* = .003, η_p_^2^ = .12, [0.03, 0.21]. Subsequent analyses explored the overshadowing effect in each group and level of reinforcement.

We compared the overshadowing index in each category with 0. One-sample *t* tests revealed than in Group 1s, there was no overshadowing considering nonreinforced cues in the color category, *t*(31) = 0.10, *p* = .921, *d* = 0.02, (*BF*_01_ = 5.27) but there was in the symbol category, *t*(31) = 4.25, *p* < .001, *d* = 0.75. The same pattern was observed in the reinforced cues, with no overshadowing in the color, *t*(31) = 1.45, *p* = .156, *d* = 0.26, (*BF*_01_ = 2.04), but reliable overshadowing in the symbol, *t*(31) = 2.85, *p* = .008, *d* = 0.50. Overshadowing was present in the symbol category, notably such competition was mitigated in the most salient category, the color.

In the case of Group 3s, in the nonreinforced cues, there was no overshadowing in neither category, *t*(31) = 1.56, *p* = .128, *d* = 0.28, (*BF*_01_ = 1.76) and *t*(31) = 1.19, *p* = .243, *d* = 0.21, (*BF*_01_ = 2.77) for color and symbol categories, respectively. In the case of reinforced cues, we observed overshadowing in the color, *t*(31) = 3.13, *p* = .004, *d* = 0.55, but not in the symbol, *t*(31) = 1.66, *p* = .106, *d* = 0.11, (*BF*_01_ = 1.54).

Finally, in the case of Group 9s for nonreinforced cues, overshadowing was reliable in the color category, *t*(34) = 4.98, *p* < .001, *d* = 0.84, but not in the symbol category, *t*(34) = 1.41, *p* = .168, *d* = 0.24, (BF_01_ = 2.23). Moreover, for reinforced cues we observed overshadowing in both categories, *t*(34) = 3.31, *p* = .002, *d* = 0.56 and *t*(34) = 2.90, *p* = .007, *d* = 0.49, for color and symbol, respectively.

#### Rule Stated

Additional analyses were conducted splitting participants by their declared rule. Table 3 shows the number of participants in each of the experiments reported here that declared using the strategy “both” (participants used color and symbol), the strategy “color” (participants only used colors), or the strategy “symbol” (participants only used symbols). In the present Experiment 1, most participants declared using both cues (*n* = 58), followed by color (*n* = 33) and only a small proportion of participants reported using the symbol (*n* = 8).[Table tbl3]

Panels C and D in Figure 3 display the overshadowing index across the three groups based on the declared processing strategy. A 3 (rule: both, color, symbol) × 2 (reinforcement: reinforced vs. nonreinforced) × 2 (category: color vs. symbol) mixed-ANOVA revealed a main effect of reinforcement, *F*(2, 96) = 36.93, *p* < .001, η_p_^2^ = .43, 90% CI [0.30, 0.52]. None other effect was significant, largest *F* for the interaction Category × Rule, *F*(2, 96) = 2.86, *p* = .062. However, a visual inspection of the reinforced cues suggests an asymmetrical overshadowing depending on participants’ choice of strategy to solve the task. For the subgroup color, overshadowing was attenuated in the category color; that is, the color cue reinforced in compound (i.e., target) and the control color cue trained alone received similar ratings during the test (middle bar in panel C), *t*(32) = 1.25, *p* = .221, *d* = 0.22, (*BF*_01_ = 3.45). On the contrary, for the symbol category, the symbol cue trained by itself received higher ratings than the symbol cue trained in compound, as suggested by the overshadowing index clearly above zero (middle bar in panel D), *t*(32) = 2.89, *p* = .002, *d* = 0.50. Although the opposite pattern was observed for subgroup symbol (right bars of panels C and D), in both cases this index was not different from zero, *t*(7) = 2.33, *p* = .053, *d* = 0.82, (*BF*_01_ = 0.54) for the color category and *t*(7) = 1.15, *p* = .285, *d* = 0.41, (*BF*_01_ = 1.76). However, as noted by the Bayes factor, in both cases the evidence for the null effect was weaker, especially in the case of color category. The lower number of participants selecting this strategy made difficult to obtain a conclusive pattern of results. The asymmetric overshadowing was not evident in the nonreinforced cues.

### Discussion

In this experiment, a reliable overshadowing effect was observed in both categories. In general, the color category worked better than the symbol category as a predictor either for the presence or the absence of the outcome. The higher ratings observed in the color category seem to indicate that the color was easier to learn about than the symbol category. Using this rate of learning as a proxy for salience, it suggests that color acts as a more salient category than symbol. However, this unbalanced salience had no clear impact on the magnitude of overshadowing.

Despite Group 9s having roughly 9 times longer exposure to the cues than Group 1s, this did not result in a reduced overshadowing effect as expected (cf., Sissons et al., 2009). However, the salience of the two categories (color and symbol) seems to have balanced with prolonged stimulus duration. At a performance level, there was a clear bias toward the color category in the Group 1s: this group did not show overshadowing in the color category, neither in the nonreinforced or the reinforced cues, suggesting that with a very short exposure participants may be focused in processing only color cues—and ignore the symbol. In line with this idea, it would be reasonable to expect that most of the participants in Group 1s would have chosen “color” as their rule; however, we observed that “both” was the preferred rule in this group.

A tendency toward asymmetric overshadowing was observed after splitting the sample by the rule stated, but only in participants who selected to process either color or symbol. Participants that declared using the color showed attenuated overshadowing during the test for the color category, but not for the symbol category. To some extent, the opposite pattern was observed in the group symbol. Although this pattern only seems reliable in the reinforced cues, it suggests that when participants freely process a particular category of the compound, they tend to disregard the alternative category, showing similar learning to the cue trained in compound and the control cue trained alone in that particular category. However, the preference to process both categories did not translate into reduced overshadowing for neither category, as expected from a pure competition perspective when trained with multiple cues. This remains true even when participants were exposed to the cues for a considerable amount of time (Group 9s). Nevertheless, it could be argued that the participants’ statement of using both categories, or only one, does not necessarily reflect that they are truly processing the cues in that way. To overcome this last issue, in Experiment 2, we administered pretraining with a biconditional discrimination—that force participants to attend to both categories—or pretraining with only one category being relevant to solve the discrimination—either the color or the symbol. In this way, we presumably manipulated the predictiveness—and the salience—of each category through the prior experience of the participants resolving a discrimination, enhancing the relevance of a given category in line with the predictiveness principles (Mackintosh, 1975). The potential effect of the pretraining was tested with new stimuli of both categories using a within-subjects overshadowing design as that used in Experiment 1.

## Experiment 2

Before training in the overshadowing task, participants in Experiment 2 were randomly assigned to one of three groups: Biconditional, Color-Relevant, and Symbol-Relevant. Participants in group Biconditional were given pretraining with a set of stimuli different to the ones to be used in the overshadowing task; these stimuli, however, belonged to the same categories used during the overshadowing training: color and symbol as described in Experiment 1. Group Biconditional was therefore trained in the following discrimination task: F**T**+, F**U**−, G**T**−, G**U**+, where F and G were colors and T and U were Chinese symbols. This discrimination task makes irrelevant the elements of the compounds (F is reinforced in the presence of T, but not in the presence of U; T is reinforced in the presence of F but not in the presence of G, etc.). When a compound is presented, the combination of both cues is needed to successfully predict the outcome. This configuration should equal the salience of each category, resulting in similar acquired salience. Note that this previous experience can play a similar role to the lengthy exposure in Group 9s of Experiment 1, in which longer time of exposure to the compound resulted in similar ratings between the elements of the compound. However, although the long exposure was insufficient to reduce overshadowing in Experiment 1, it suggests that the processing of both cues of the compound was similar.

Two other groups were given pretraining where only one category (either color or symbol) was relevant. Color-Relevant and Symbol-Relevant groups were exposed during pretraining to the same four compounds as the Biconditional group; however, only one category was relevant to solve the discrimination, either the color or the symbol (see Table 4). The expectation was that training participants to attend to one of the categories would result in asymmetric overshadowing—as suggested by the results of Experiment 1, in which participants that declared using just one category showed a tendency to attenuated overshadowing in the selected category.[Table tbl4]

We are not aware of any studies that apply this category-relevant pretraining to overshadowing other than studies in the spatial learning domain by Buckley et al. (2014, 2015). The logic of this manipulation was that making relevant a given category (e.g., color) in a previous stage may transfer this information to the subsequent overshadowing discrimination, using a different set of stimuli belonging to the same categories (see Urcelay & Miller, 2010). That is, in the Color-Relevant group, for example, the color cues would be prioritized if the transfer of information is adequate, and this may result in the alternative category not being fully processed. Finally, given that we observed a large variance in the overshadowing index in the previous experiments, especially when splitting the sample by their declared rule (with a very low number of participants declaring using the symbol category), in Experiment 2 we doubled the sample of participants per group to reduce the observed variability and increase the sensitivity of the statistical tests.

### Method

#### Participants

Two hundred forty participants (106 female, 132 male, one nonbinary, and one preferred not to say) were recruited from Prolific. Their mean age was 42.19, *SD* = 15.01 (range 18–81). Participants received £2.50 as compensation. The study was approved by the Ethics Committee at the University of Nottingham.

#### Design and Procedure

The design of Experiment 2 is displayed in Table 4. The overshadowing and test phases were identical to Experiment 1. For the pretraining phase, we used two new colors, orange (244, 183, 0) and gray (191, 191, 191), and two new symbols, frog [蛙] and fox [狐]. These stimuli were counterbalanced across subjects. The Biconditional group received a biconditional discrimination as pretraining. That is, the compounds FT and GU were always reinforced whereas the compounds FU and GT were never followed by reinforcement. The color and symbol cues used during overshadowing were the same as in Experiment 1. In the Color-Relevant group, one color was associated with the outcome (F) while the second color (G) was associated with the absence of the outcome, whereas the symbols (T and U) were equally associated with the outcome and its absence, making them irrelevant to solve the task. The same logic applied to the Symbol-Relevant group, making the symbols relevant and the colors irrelevant (Table 4).

Unlike the previous experiment, time constraints during training were removed. Hence, the stimuli appeared for 1s on the screen, and then the question appeared with the stimuli still on the screen. After the question appeared, participants could take as much time as they wished to make their response before receiving feedback. All other procedural details replicated the previous experiment. The pretraining stage was divided into two stages. In the first stage, there were 12 pretraining trials of each compound. However, after the sixth block of training (12 trials of each cue), the pretraining trials were intermixed with the overshadowing training trials. That is, in the second stage, pretraining and overshadowing training overlapped to improve the transfer from the pretraining to the overshadowing stages (see Williams et al., 1994).

#### Data Analyses

For the sake of clarity, only participants that matched their pretraining with their declared rule were analyzed. That is, for group Biconditional, we only considered participants who declared using both cues during the task (similar to participants who selected “both” at the end of Experiment 1); for group Color-Relevant, only participants who declared using the color and for the group Symbol-Relevant the participants who selected symbol. In this way, we analyzed participants whose declared strategy matched the pretraining they received. We expected a higher proportion of participants in group Biconditional to choose “both,” in group Color-Relevant to choose “color” and in group Symbol-Relevant to choose “symbol.” Table 3 confirmed this distribution: 89% of participants pretrained in the biconditional task declared using both the color and the symbol, indicating that the pretraining was successful at promoting attention to both stimulus dimensions; 75% of participants trained in the color-relevant discrimination declared using the color; finally, 61% of participants trained in the symbol-relevant discrimination declared using the symbol. After applying the inclusion criteria detailed in Experiment 1, plus the matching of group and rule stated here, we analyzed 58, 44, and 40 participants in groups Biconditional, Color-Relevant, and Symbol-Relevant, respectively.

### Results

#### Pretraining

**Figure 4 fig4:**
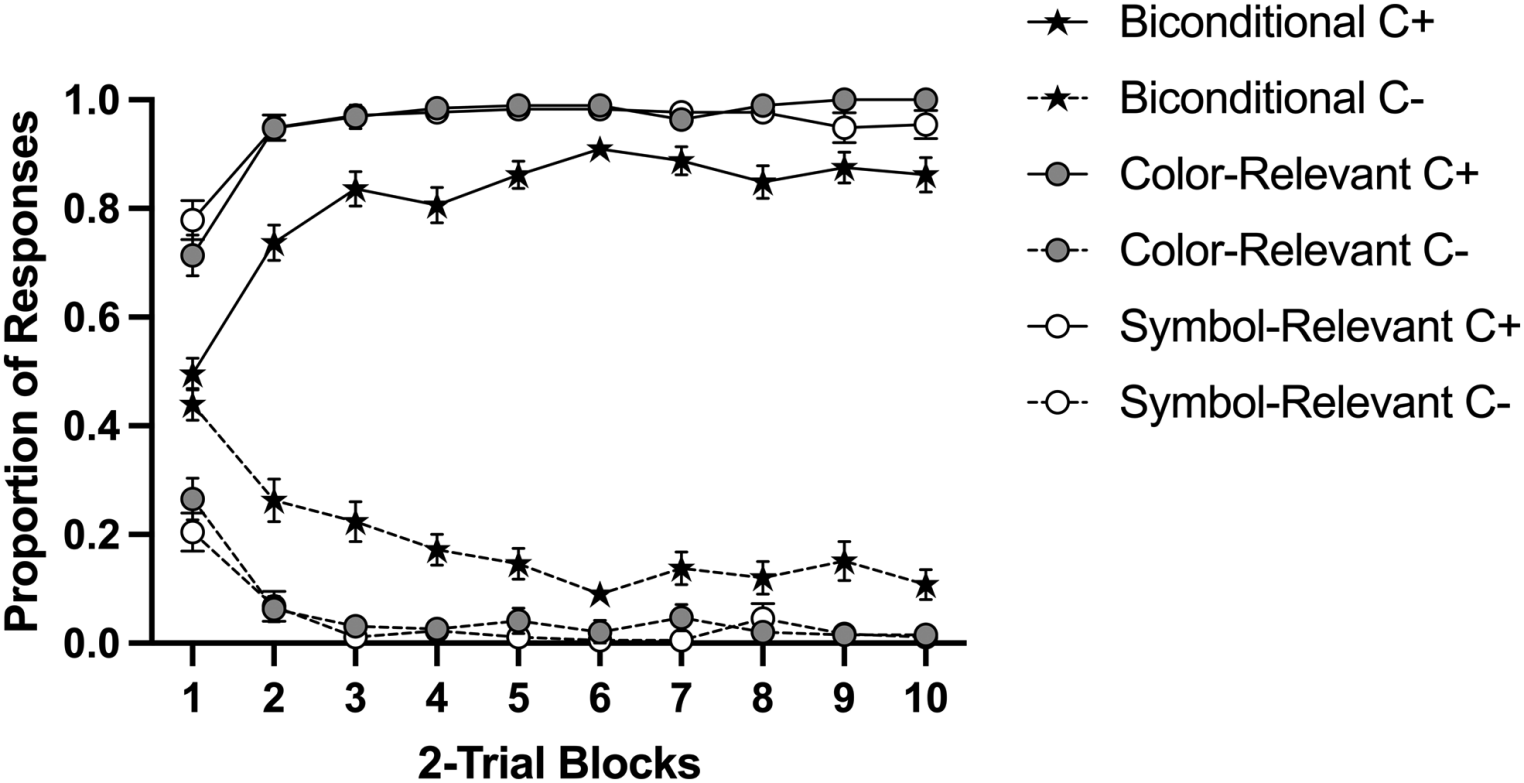
Pretraining Discrimination in Experiment 2 *Note*. Proportion of participants who predicted the shock outcome in blocks of two trials. Star symbols symbolize group Biconditional, gray symbols group Color-Relevant and white symbols group Symbol-Relevant. Solid lines represent the reinforced cues and dashed line represents the nonreinforced cues. Error bars are *SEM*.

For the sake of simplicity, we collapsed compounds according to their reinforcement value (reinforce compound C+; nonreinforced compound C−). A mixed-ANOVA with group, reinforcement (C+ vs. C−), and blocks of trials revealed a significant three-way Group × Reinforcement × Block interaction, *F*(18, 1323) = 4.18, *p* < .001, η_p_^2^ = .05, 90% CI [0.02, 0.06]. As Figure 4 suggests, group Biconditional acquired the discrimination slower than the other two groups; however, the discrimination between the reinforced and nonreinforced compounds was clear in all groups. In the last block of training, the main effect of group was significant for reinforced compounds (C+), *F*(2, 147) = 8.51, *p* < .001, η_p_^2^ = .10, 90% CI [0.04, 0.18]. Tukey post hoc revealed differences between Biconditional compared to Color-Relevant, *p* < .001 and Symbol-Relevant, *p* = .026. However, there were no differences between Color-Relevant and Symbol-Relevant, *p* = .433. The same pattern was found in the nonreinforced cues, *F*(2, 147) = 10.03, *p* < .001, η_p_^2^ = .12, 90% CI [0.04, 0.20]. Again, Tukey post hoc revealed differences between Biconditional compared to Color-Relevant, *p* < .001 and Symbol-Relevant, *p* = .001, and these did not differ from each other *p* = .999.[Fig fig4]

#### Overshadowing training

**Figure 5 fig5:**
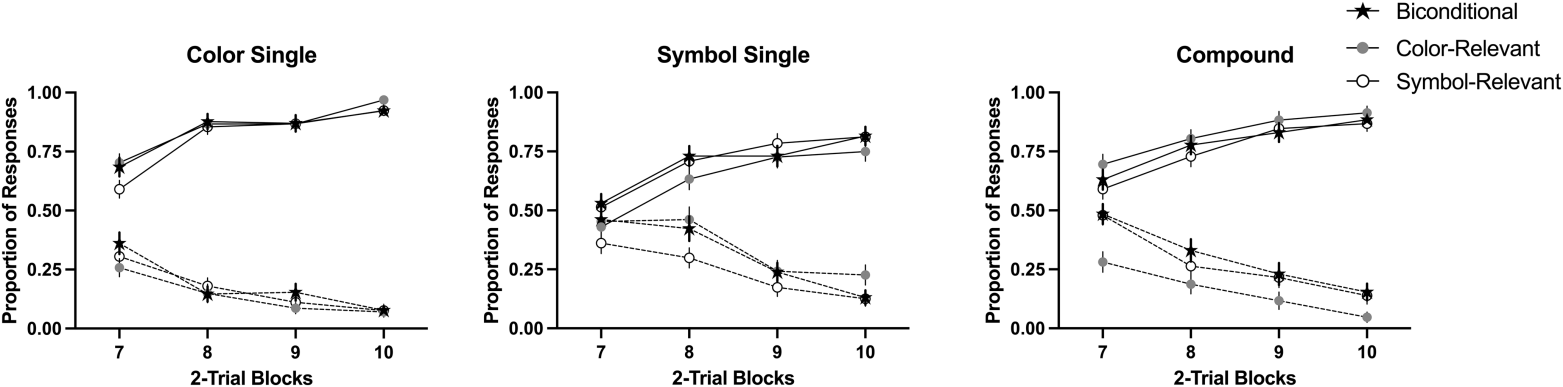
Overshadowing Discrimination in Experiment 2 *Note*. Proportion of participants who predicted the shock outcome in blocks of two trials. Each figure represents the response in the presence of the color cue alone (left), the symbol cue alone (middle), and the compound of color + symbol (right). Star symbols symbolize group Biconditional, gray symbols group Color-Relevant and white symbols group Symbol-Relevant. Solid lines represent the reinforced cues and dashed line represents the nonreinforced cues. Error bars are *SEM*.

As in the previous experiment, we focused on the last block of training. A 3 (group: biconditional, color-relevant, symbol-relevant) × 2 (reinforcement: reinforced vs. nonreinforced) × 3 (type: color, symbol, compound) ANOVA revealed a main effect of reinforcement *F*(1, 147) = 1,821.85, *p* < .001, η_p_^2^ = .92, 90% CI [0.90, 0.94], and a significant triple interaction, *F*(4, 294) = 4.75, *p* = .001, η_p_^2^ = .06, [0.02, 0.10]. Subsequent analyses of this interaction were conducted at each level of reinforcement. In the case of the reinforced cues, there was a significant effect of type, *F*(2, 294) = 10.54, *p* < .001, η_p_^2^ = .07, 90% CI [0.03, 0.11], but not of group, *F*(2, 147) = 0.27, *p* = .762, nor an interaction between group and type, *F*(4, 294) = 1.86, *p* = .117. Despite all groups performing similarly, and in line with the previous experiment, the overall response to the symbol was lower compared to the color category, *F*(1, 149) = 17.18, *p* < .001, η_p_^2^ = .10, 90% CI [0.04, 0.18] and the compound, *F*(1, 149) = 8.76, *p* = .004, η_p_^2^ = .05, [0.01, 0.12]. Similarly, for the nonreinforced cues, the effect of type was also significant, *F*(2, 294) = 10.54, *p* < .001, η_p_^2^ = .07, [0.03, 0.11], but neither the interaction Group × Type nor the effect of group was significant, largest *F*—for the interaction, *F*(4, 294) = 1.86, *p* = .117. A higher rate of responding was observed to the symbol cues compared to the color cues, *F*(1, 149) = 6.99, *p* = .009, η_p_^2^ = .04, 90% CI [0.01, 0.11], but not to the compound, *F*(1, 149) = 2.05, *p* = .154. In short, the performance to the symbol cues was poorer compared to the other two types of cues (Figure 5).[Fig fig5]

#### Test Phase

As expected, Table 5 shows good discrimination according to the value of the cues in the previous stage: high ratings to reinforced cues, low ratings to nonreinforced cues and intermediate values to partially reinforced cues. Moreover, as in the previous experiment, we evaluated the response to the target cues, that is, we compared the response to the elements forming the compound. In the reinforced cues, there was a main effect of category, with color receiving overall higher ratings than the symbol category, *F*(1, 147) = 6.89, *p* = .010, η_p_^2^ = .04, 90% CI [0.01, 0.11]; however, this effect was modulated by group, *F*(2, 147) = 11.93, *p* < .001, η_p_^2^ = .14, [0.06, 0.22]. In group Biconditional target cues from both categories received similar ratings, *t*(57) = 1.16, *p* = .249, (*BF*_01_ = 3.67). Notably, color category received higher rating than symbol category in group Color-Relevant, *t*(47) = 5.43, *p* < .001, and the opposite pattern was found in the Symbol-Relevant group, with symbol category recruiting higher level of anticipation of the outcome relative to the color category, although this difference was at the threshold to be significant, *t*(43) = 1.94, *p* = .059. A similar pattern emerged with the nonreinforced cues, in which the interaction Category × Group was significant, *F*(2, 147) = 9.75, *p* < .001, η_p_^2^ = .11, 90% CI [0.04, 0.19], but not the main effect of category, *F*(1, 147) = 1.20, *p* = .275. Again, there were no differences between both categories in group Biconditional, *t*(57) = 0.57, *p* = .569, (BF_01_ = 5.96), but there were differences in the Color-Relevant, with color receiving lower ratings than symbols, *t*(47) = 4.04, *p* < .001, and the opposite was true in the case of Symbol-Relevant group, with symbol cue receiving lower outcome expectancy than color *t*(43) = 2.36, *p* = .023.[Table tbl5]

**Figure 6:**
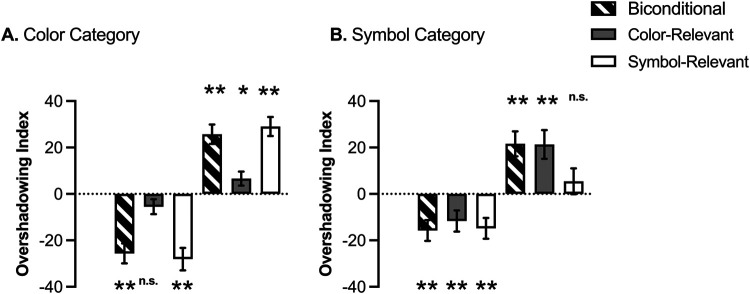
Overshadowing Index in Experiment 2 *Note*. Panel A displays the overshadowing indexes for the color cues whereas Panel B displays the indexes for the symbol cues. Note that the overshadowing index is calculated by subtracting the predictive rating of the target cue (trained in compound with a competitor) from the ratings of the control cue (trained by itself); a value of 0 represents similar ratings to the control and target cues, that is, no overshadowing. Positive values are indicative of overshadowing. * *p* < .005. ** *p* < .001. n.s. *p* > .05. Error bars are *SEM*.

Figure 6 reveals that group Biconditional showed similar overshadowing in both categories. However, the other two groups showed an asymmetric pattern. For the reinforced cues, in group Color-Relevant overshadowing was attenuated in the color category, while the opposite pattern was true in the case of group Symbol-Relevant. For the nonreinforced cues, the pattern was more ambiguous. A 3 (group: biconditional, color-relevant, symbol-relevant) × 2 (reinforcement: reinforced vs. nonreinfroced) × 2 (category: color vs. symbol) mixed-ANOVA revealed a three-way interaction, *F*(2, 147) = 8.61, *p* < .001, η_p_^2^ = .10, 90% CI [0.03, 0.18], superseding the rest of effects and interactions.^1^

To evaluate the reliability of overshadowing, one-sample *t* tests compared the overshadowing index with 0 in each group, category, and level of reinforcement. In the case of Biconditional group, for the nonreinforced cues, overshadowing was significant in both categories: color, *t*(57) = 5.93, *p* < .001, *d* = 0.78, and symbol, *t*(57) = 3.49, *p* = .001, *d* = .46. Moreover, a direct comparison between both categories (paired-sample *t*), did not reveal differences between them, *t*(57) = 1.65, *p* = .106 (*BF*_01_ = 1.97). For the reinforced cues, a similar pattern emerged, with an index over zero in the color, *t*(57) = 6.13, *p* < .001, *d* = 0.81 and symbol, *t*(57) = 4.03, *p* < .001, *d* = 0.53 categories, without differences between both categories, *t*(57) = 0.57, *p* = .567, *d* = 0.07 [*BF*_01_ = 5.95], suggesting a similar magnitude of overshadowing.

In the case of group Color-Relevant, for the nonreinforced cues overshadowing was not present for the color category, *t*(47) = 1.71, *p* = .093, (*BF*_01_ = 1.64]) but it was reliable in the symbol category *t*(47) = 2.56, *p* = .014, *d* = 0.37, although there were no differences between them, *t*(47) = 1.12, *p* = .272 (*BF*_01_ = 3.57). However, overshadowing was reliable in both categories with the reinforced cues, for the color *t*(47) = 2.20, *p* = .033, *d* = 0.32 and symbol *t*(3.44) = 3.40, *p* = .001, *d* = 0.50, respectively, however, the magnitude of overshadowing was weaker in the color compared to symbol category, *t*(47) = 2.27, *p* *=* .028, *d* = 0.33.

In the case of Symbol-Relevant, overshadowing was reliable in both categories with the nonreinforced cues, *t*(43) = 5.81, *p* < .001 and *t*(43) = 3.30, *p* = .002, for color and symbol, respectively. However, the magnitude of overshadowing was lower with symbol compared to color, *t*(43) = 2.11, *p* = .041. In the case of reinforced cues, overshadowing was significant for the color *t*(43) = 7.11, *p* < .001, *d* = 1.07 but not for symbol *t*(43) = 0.97, *p* = .336, *d* = 0.14 (*BF*_01_ = 3.93); moreover, there was a significant difference between the magnitude of overshadowing in both categories, *t*(43) = 3.85, *p* < .001, *d* = 0.58, with attenuated overshadowing in the symbol category.

### Discussion

When one category was made relevant during pretraining, either color or symbol (and participants declared to have used that category), there was reduced overshadowing of that category compared with the alternative category. Despite the general tendency leading to overshadowing with this preparation and parameters, competition was attenuated by pretraining a relevant category, but not when the pretraining encouraged processing both categories simultaneously, as was the case in group Biconditional. In the present experiment, group Biconditional learned slower and reached a lower level of discrimination (although reliable) during the pretraining than the other two groups, replicating previous findings (Livesey et al., 2019). Limited mastering of the discrimination may result in a relatively weak transfer of the processing mode to the subsequent discrimination (see Shanks & Darby, 1998), so it could be the case that group Biconditional in this experiment was more limited than the other two groups while processing both categories. However, it should also be noted that processing both categories is cognitively more demanding than only attending to one category, and this limited-resourced capacity may underlie the strong overshadowing effect observed in both categories.

## General Discussion

Across two experiments, overshadowing was observed in a predictive learning paradigm using two separable categories (colors and symbols) that can be attended independently—as opposed to integral dimensions (Garner, 1976). The color category was expected to be of greater salience than the symbol category—the color can be processed immediately whereas the symbol category requires time and effort to process. This a priori assumption was supported by the fact that the color category was readily learned about when compared to the symbol category. In Experiment 1, with short compound stimulus (color and symbol) duration (1s), we observed a nonreciprocal overshadowing: the symbol failed to overshadow the color cue whereas the color successfully overshadowed the symbol. Longer compound stimulus duration (9s) resulted in similar ratings of the elements of the compound—suggesting similar salience of both categories; however, overshadowing was still quite robust when compared with the corresponding control cues. Experiment 2 showed that enhancing the salience of a category (either color or symbol) protects it from overshadowing—that is, if the category color is trained as relevant to predict an outcome, the symbol fails to subsequently overshadow the color (and vice versa). Additionally, participants trained in a biconditional discrimination that promotes the joint use of the color and the symbol yielded similar ratings between the elements integrating the compound, suggesting the salience of both categories was similar; however, this was not enough to attenuate overshadowing when compared to the cues trained in isolation in the control condition.

As mentioned in the introduction, there are few examples of cue competition studies with humans in which the relative salience of the elements of a compound was manipulated. There are some instances of blocking experiments with contrasting outcomes: one study revealed that increasing the salience of the blocked cue protects it from blocking (Denton & Kruschke, 2006) and another showing the opposite, that is enhanced blocking when using a highly salient blocked (or target) cue (Le Pelley et al., 2014). Our studies provide a new piece of evidence in this line of research focusing on the overshadowing effect, suggesting that the relative salience of the elements of the compound has no clear effect on the magnitude of competition (also see Murphy & Dunsmoor, 2017). Despite the fact that we observed an advantage of the color compared to the symbol cues during training and more precise judgments during the test phase, we did not observe an effect of category in the magnitude of overshadowing. That is, overshadowing was usually quite robust regardless of the salience of the category, and only attenuated when a particular category was made predictive during pretraining in Experiment 2. This result is at odds with several studies in animals (e.g., Mackintosh, 1976; Exp 2), which found that when the two elements of the compound are of high salience, they tend to attenuate the overshadowing effect. Further research seems to be needed to address the role played by the relative salience of stimuli in the case of human predictive learning.

An interesting outcome of this study is that when participants experienced long exposure to the compound stimuli (Group 9s in Experiment 1) or pretraining with a biconditional discrimination (group Biconditional in Experiment 2) the ratings to both elements of the compound were alike. This pattern suggests that the salience of both categories was similar, yielding similar control on the overall prediction. However, this was insufficient to reduce overshadowing when compared to the control cues trained alone. As mentioned in the *Introduction*, when the two elements of the compound are of high salience, overshadowing is sometimes mitigated (Mackintosh, 1976). It could be argued that these two manipulations (long exposure to the compound cue and pretraining carried out to improve the attention to the two elements of the compound) were not sufficient to boost the salience of each category to the required level to observe an attenuation of cue competition. The fact that the comparison was conducted at the same time with the two dimensions in a within-subjects design may have resulted in a very challenging situation which undermined this potential effect. However, previous studies did not find differences in the magnitude of blocking as a function of the complexity of the design (Vandorpe & De Houwer, 2006), so this conclusion is speculative at the moment.

According to the acquired predictiveness principle (Le Pelley et al., 2016; Mackintosh, 1975), the stimuli of a category perceived as relevant should recruit attention at the expense of other categories perceived as less relevant. In Experiment 2, for example, during the overshadowing training, participants in the Color and Symbol-Relevant groups were trained with a new cue that belonged to a relevant category and another new cue that belonged to an irrelevant category. The increased attention to the relevant category can be expected to gradually increase the associability of the new cues belonging to that category as the overshadowing training progresses whereas the associability of the irrelevant category can be expected to decrease. This rationale fits with the results observed in Experiment 2—nonreciprocal overshadowing following training that boosts the salience of one category—and resemble the intradimensional versus extradimensional shift effects (ID vs. ED) but applied to a cue competition scenario. There is compelling evidence that intradimensional training facilitates subsequent discriminations using new stimuli of that particular category (e.g., Durlach & Mackintosh, 1986; Mackintosh & Little, 1969; Roberts et al., 1988). However, as mentioned above, this type of manipulation has been rarely assessed in the cue competition domain with the exception of one example in blocking (Kaminski et al., 2008) and in the spatial domain (Buckley et al., 2014, 2015). On the other hand, in the case of the biconditional discrimination, both categories should have been relevant to predict the outcomes during pretraining. At the time of the overshadowing training with two new cues, that belong to relevant categories we expected both cues to be able to overshadow each other, resulting in reciprocal overshadowing. It is worth mentioning that the control cues trained in isolation are likely to recruit high levels of attention and therefore acquire strong predictive value by themselves.

In the case of group Biconditional, the pretraining may have induced a stronger configural processing (e.g., Glautier et al., 2016). Previous studies have observed that configural pretraining attenuated overshadowing in rodents (e.g., Urcelay & Miller, 2009) and blocking in humans (e.g., Williams et al., 1994). However, we did not observe this pattern in Experiment 2, suggesting that our task setting may not be sensitive to these manipulations. Previous rodent studies have used different cues along the same sensory modality (e.g., tone and clicker). While we also used stimuli from the same sensory modality (i.e., visual), they belonged to different categories: colors and symbols. Across experiments, color cues consistently received higher ratings than symbol cues during training and test, suggesting that in general color cues are more readily associated with the outcome than symbols. Note that this result is not surprising, since we anticipated that the color category might be easier to learn about. Unbalanced salience between stimuli has been shown to impair learning of biconditional discrimination in humans (Byrom & Murphy, 2019), and consequently, it could be the case that our stimuli, by virtue of their unequal saliences, prevented the transfer a configural processing to overshadowing training in group Biconditional. Additionally, other studies have shown that transfer effects enhancing configural processing were only reliable on the very first block of training of a subsequent discrimination (Mehta & Russell, 2009), suggesting that in general the transfer of the configural pretraining may be dependent upon very specific parameters of training or may be short lived (see Urcelay & Miller, 2010; Wheeler et al., 2008). Moreover, recent modeling studies using a multilayered connectionist network suggest that the biconditional discrimination can be solved by either elemental or configural solutions (see Castiello et al., 2021), and therefore it is possible that participants solved the biconditional discrimination by other means. Therefore, it is unclear that biconditional discrimination pretraining resulted in configural encoding of the information, making it less likely to reduce the overshadowing effect during overshadowing training.

Having said that, the result of Experiment 2 can be integrated into an attentional configural theory, in which the previous relevance of a dimension is taken into account. George and Pearce (2012) proposed a modification to Pearce’s (1987, 1994) configural theory that incorporates how changes in attention driven by predictiveness may explain the ID versus ED phenomenon. They proposed that stimuli of the same category may activate a common receptor unit. Such a common receptor unit is presumably activated by stimuli varying along that category (e.g., color or orientation). If that category has acquired relevance, it is supposed to receive more attention and consequently increase the salience of the stimuli in that category. They assumed that this sharing unit could trigger larger generalization between stimuli of the same category, resulting in the facilitation effect observed in ID versus ED shifts (e.g., George & Pearce, 1999). The extension of such interpretation to our data seems clear. Making relevant a particular category (e.g., color) during pretraining increased the salience of the stimuli of that particular category during the overshadowing training, attenuating the competition from other stimuli (e.g., symbol) usually observed in that scenario. This presumably results in weaker generalization decrement from the training compound to the test stimulus (Pearce, 1987, 1994). Thus, these results seem in line with interpretations based on attentional and configural mechanisms of learning. However, pretraining concurrently both categories with the biconditional discrimination did not attenuate overshadowing, suggesting that the effect is only reliable when pretraining a particular category. In general, our data suggest that the previous experience with the relevance (or irrelevance) of a whole category modulated subsequent overshadowing.

One important limitation of our study is that salience was not explicitly controlled. Despite the fact that we provided several reasons to justify that color would be a more salient category and the facilitation observed in the rate of learning seems to confirm that, we did not manipulate salience in a controlled way (as in, increasing the physical intensity of the stimuli). In this line, a more accurate manipulation of salience varying along the same dimension (e.g., Le Pelley et al., 2014 with the semantic salience) may provide a better scenario to explore the specific contribution of salience in the magnitude of overshadowing. However, our use of separable categories also provided an interesting set of results in terms of relative salience. Relatedly, an unexplored issue of this experimental series refers to the specific mechanism recruited during overshadowing training. As mentioned above, we speculated that the results in Experiment 2 are due to an increase of attentional resources to the predictive category. However, it could also be attributable to a reduction in the processing of the irrelevant category. In our experiments, it is difficult to assess such an approach, because one category (symbol) is presented over the other, and consequently, both stimuli share the same visual space. Future studies may want to address this issue, evaluating the tradeoff between the relevance (or irrelevance) of a particular category and the fate of cue competition phenomena.

Although competition is the most likely outcome following compound conditioning, several manipulations can attenuate or even reverse this outcome resulting in potentiation (e.g., Alcalá, Kirkden, et al., 2023; Cunha et al., 2015; Urcelay & Miller, 2009), highlighting the need to identify variables that are likely to affect cue interaction phenomena. In this experimental series, we observed that competition depends to some extent on the relative salience of the elements forming the compound. This salience was subtly modified by the time of exposure to the cues in Experiment 1 and was clearly modified by the previous experience with a particular category trained as relevant in a discrimination task in Experiment 2. This last result opens an interesting avenue to further explore the role played by prior experience in cue-competition phenomena.

## Figures and Tables

**Table 1 tbl1:** Design of Experiment 1

Group	Overshadowing	Test
1s	8A+, 8B−, 8**V**+, 8**W−**, 8C**X**+, 8D**Y−**, 8E±, 8**Z**±	A? B? C? D? E? **V? W? X? Y? Z?**
3s		
9s		
*Note*. Letters A–E represent different colors and Letters V–Z (in bold) represent different symbols. “+” symbolizes the outcome, “−” represents the absence of outcome, “±” represents partial reinforcement; “?” symbolizes the test question without feedback. Numbers indicate how many trials were run per cue. The key comparison was between A and C (colors trained by themselves or in compound with the symbol X, respectively) and between V and X (symbols trained by themselves or in compound with the color C).		

**Table 2 tbl2:** Averaged Ratings During Test Phase of Experiment 1

Group	Stats	Colors							Symbols						
		A+	C+	B−	D−	E±	OVIndex (Non)	OV Index (Rein)	V+	X+	W−	Y−	Z±	OVIndex (Non)	OVIndex (Rein)
1s	*M*	91.8	81.8	11.6	12.2	53.1	−0.59	10.0	74.1	52.7	19.2	42.9	63.5	−23.69	21.4
	*SD*	20.5	28.8	23.9	24.7	32.4	33.40	38.9	28.5	33.0	22.9	31.8	20.0	31.51	42.6
3s	*M*	81.2	66.7	22.0	30.6	59.3	−8.62	14.5	66.5	56.3	32.3	40.0	54.6	−7.66	10.2
	*SD*	26.2	29.7	26.8	29.2	25.5	31.17	26.2	30.5	27.4	31.8	25.8	25.2	36.35	34.6
9s	*M*	83.8	65.2	10.0	33.2	56.7	−23.2	18.7	82.7	65.4	22.7	29.4	61.3	−6.65	17.3
	*SD*	28.0	27.5	16.2	27.7	27.9	27.52	33.4	22.3	28.3	24.9	27.7	28.0	27.96	35.3
*Note*. Letters A–E refer to color category, and letters V–Z refer to symbol category. The “+” means that the cue was reinforced in the previous stage; “−” means that the cue was not reinforced; “±” represents partial reinforcement. The gray columns represent that the cue was part of a compound during the training phase. OV Index is the overshadowing index (Non = nonreinforced cues; Rein = reinforced cues); positive scores are indicative of overshadowing (see text).															

**Table 3 tbl3:** Distribution of Participants by Group and Rule Used Across Experiments

Exp	Group	Both	Color	Symbol
1	1s (*n* = 32)	21	10	1
	3s (*n* = 32)	12	18	2
	9s (*n* = 35)	25	5	5
	Total per rule	58	33	8
2	Biconditional (*n* = 65)	58	3	4
	Color-Relevant (*n* = 64)	16	48	0
	Symbol-Relevant (*n* = 72)	23	5	44
	Total per rule	97	56	48
*Note*. The table displays the number of participants in each group according to the rule declared at the end of the experiment. In brackets is the *n* of each group. In Experiment 3, the shadow cells represent the participants included in the analyses, matching their training with the rule stated.				

**Table 4 tbl4:** Design of Experiment 2

Group	Pretraining	Pretraining (eight extra trials) + Overshadowing	Test
Biconditional	12F**T**+, 12F**U−**, 12G**T−**, 12G**U**+	8A+, 8B**−**, 8**V**+, 8**W−**, 8C**X**+, 8D**Y−**, 8E±, 8**Z**±	A? B? C? D? **V? W? X? Y?** E? **Z?**
Color-Relevant	12F**T+**, 12F**U+**, 12G**T−**, 12G**U−**		
Symbol-Relevant	12F**T+**, 12F**U−**, 12G**T+**, 12G**U−**		
*Note*. Letters A–G represent different colors and letters T–Z (in bold) represent different symbols. “+” symbolizes the outcome, “−” represents the absence of outcome, “±” represents partial reinforcement; “?” symbolizes the test question without feedback during the test phase. Numbers represent the number of trials per cue. Note that during the overshadowing phase, eight additional trials of pretraining (with the compounds FT, FU, GT, and GU) were administered intermixed with the overshadowing training trials (see text for rationale).			

**Table 5 tbl5:** Average Ratings During Test Phase of Experiment 2

Group		Colors							Symbols						
	Stats	A+	C+	B−	D−	E±	OV Index (Non)	OV Index (Rein)	V+	X+	W−	Y−	Z±	OV Index (Non)	OV Index (Rein)
Biconditional	*M*	91.6	65.9	8.3	33.9	49.4	−25.62	25.7	80.6	59.0	20.9	36.6	54.4	−15.73	21.6
	*SD*	19.0	29.1	18.6	30.8	30.6	32.86	31.9	27.8	27.2	26.1	23.5	26.3	34.32	40.8
Color-Relevant	*M*	91.6	84.9	5.5	11.0	52.4	−5.5	6.6	75.7	54.3	19.2	30.9	47.3	−11.67	21.3
	*SD*	19.4	27.2	13.7	20.4	27.3	22.23	20.9	30.4	28.7	28.3	27.3	32.6	31.51	42.9
Symbol-Relevant	*M*	90.3	61.2	9.7	37.8	54.6	−28.08	29.0	77.6	72.2	10.0	24.8	53.5	−14.79	5.4
	*SD*	18.9	21.6	19.0	27.2	29.9	32.02	27.1	29.2	29.4	16.1	28.6	28.4	29.67	37.0
*Note*. Letters A–E refer to color category, and letter V–Z refers to symbol category. The “+” means that the cue was reinforced in the previous stage; “−” means that the cue was not reinforced; “±” represents partial reinforcement. The gray shadow represents that the cue was part of a compound during previous phase. OV Index is the overshadowing index (Non = nonreinforced cues; Rein = reinforced cues); positive scores are indicative of overshadowing (see text).															

## Footnotes

1 This interaction was also significant if we did not match rule stated with each experimental group, *F*(2, 198) = 5.22, *p* = .006, η_p_^2^ = .05. Follow-up analyses revealed a somewhat similar pattern, but with overshadowing being present in all conditions, that is, across categories, reinforcement and group, the overshadowing index always was different from zero. Therefore, without matching participants’ pretraining to their stated rule, overshadowing was more robust. This could be interpreted as an attenuation of overshadowing by criteria selection of participants whereby matching their strategy with their experimental training was more sensitive to the salience of the relevant category.
